# Massive aortic root dilation in a Young male with previously undiagnosed acromegaly: a case report and review

**DOI:** 10.1093/omcr/omae185

**Published:** 2025-02-22

**Authors:** Mahmoud Gomaa, Hassan El-Shirbiny, Osama Elshaer

**Affiliations:** Department of Cardiovascular Medicine, Kafrelsheikh University Hospital, Kafrelsheikh 33511, Egypt; Department of Cardiovascular Medicine, Kafrelsheikh University Hospital, Kafrelsheikh 33511, Egypt; Department of Cardiovascular Medicine, Kafrelsheikh University Hospital, Kafrelsheikh 33511, Egypt; Faculty of Medicine, Kafrelsheikh University, Kafrelsheikh 33511, Egypt

**Keywords:** acromegaly, aortic root dilatation, uncommon presentation, Bentall procedure

## Abstract

**Background:**

Acromegaly is an endocrine disorder characterized by excessive growth hormone (GH) production, causing abnormal bone and tissue enlargement. Typically, it presents with distinct physical changes, but some cases may lack noticeable features and still have internal abnormalities like aortic root dilation. Though rare, a family history can raise suspicion.

**Case presentation:**

This report presents a case of a 23-year-old male with no significant medical history, who presented with severe dyspnea after a chest infection. He lacked distinct physical features of genetic or endocrinal disorders, but his uncle had acromegaly. Echocardiography revealed left ventricular hypertrophy and massive aortic root dilation. Elevated insulin-like growth factor 1 (IGF-1) confirmed the diagnosis of acromegaly. The patient underwent a Bentall procedure, and his acromegaly was managed with octreotide and regular follow-ups.

**Conclusions:**

The report emphasizes the potential genetic link, and the possibility of massive aortic root dilation associated with acromegaly.

## Introduction

Acromegaly is a rare hormonal disorder characterized by the excessive production of GH, typically due to a benign tumor in the pituitary gland. Although most cases are sporadic, in rare cases, acromegaly can run in families, suggesting genetic predisposition [1]. While it usually presents with distinct physical changes such as enlarged facial features or extremities, it can sometimes lack typical clinical manifestations because of slow progression over many years, so early changes might be subtle and go unnoticed.

Acromegaly can be associated with cardiovascular complications such as aortic root dilation, which is not uncommon, but is typically mild and does not require surgical intervention. In this report, we present a case of a previously healthy middle-aged man with a family history of acromegaly (his uncle). He presented with symptoms of congestive heart failure following a recent chest infection. Diagnostic tests confirmed acromegaly, and echocardiography uncovered a significantly dilated aortic root, resulting in severe aortic regurgitation and impaired left ventricular function.

## Case report

This is a case of a 23-year-old male with an unremarkable medical history who was referred with unexplained dyspnea. Prior to his current illness, he was in good health and had no difficulty performing daily activities. Both of his parents had diabetes, and his grandfather passed away from a heart attack at the age of 74. Additionally, upon further review of his family history, we discovered that the patient’s uncle has a history of acromegaly.

The patient’s condition started 2 weeks prior to admission when he started complaining of fever, nasal congestion, and rhinorrhea. Three days later, he started complaining of vomiting, diarrhea, and abdominal pain. After being admitted to the internal medicine ward, we were consulted for marked palpitations, dyspnea, and orthopnea.

During the examination, his temperature was slightly elevated at 37.9°C, blood pressure of 150/90 mmHg, heart rate was 110/min, and respiratory rate was 22/min. Auscultation revealed fine basal lung crepitations with bilateral mild expiratory rhonchi. Additionally, an early diastolic murmur was detected. His neck veins were congested, yet not pulsating, and there was no lower limb edema or ascites.

**Figure 1:**
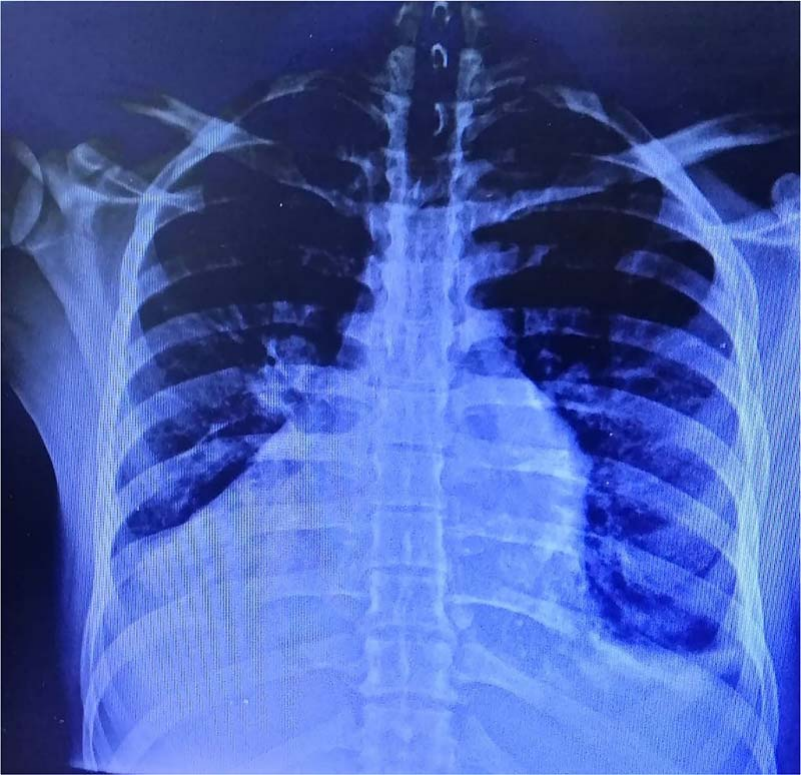
Chest X-ray showing bilateral mild pleural effusion and cardiomegaly.

**Figure 2:**
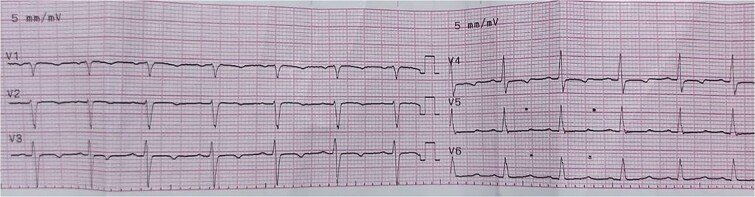
ECG showing sinus tachycardia with inverted T waves in leads V3 and V4.

**Figure 3:**
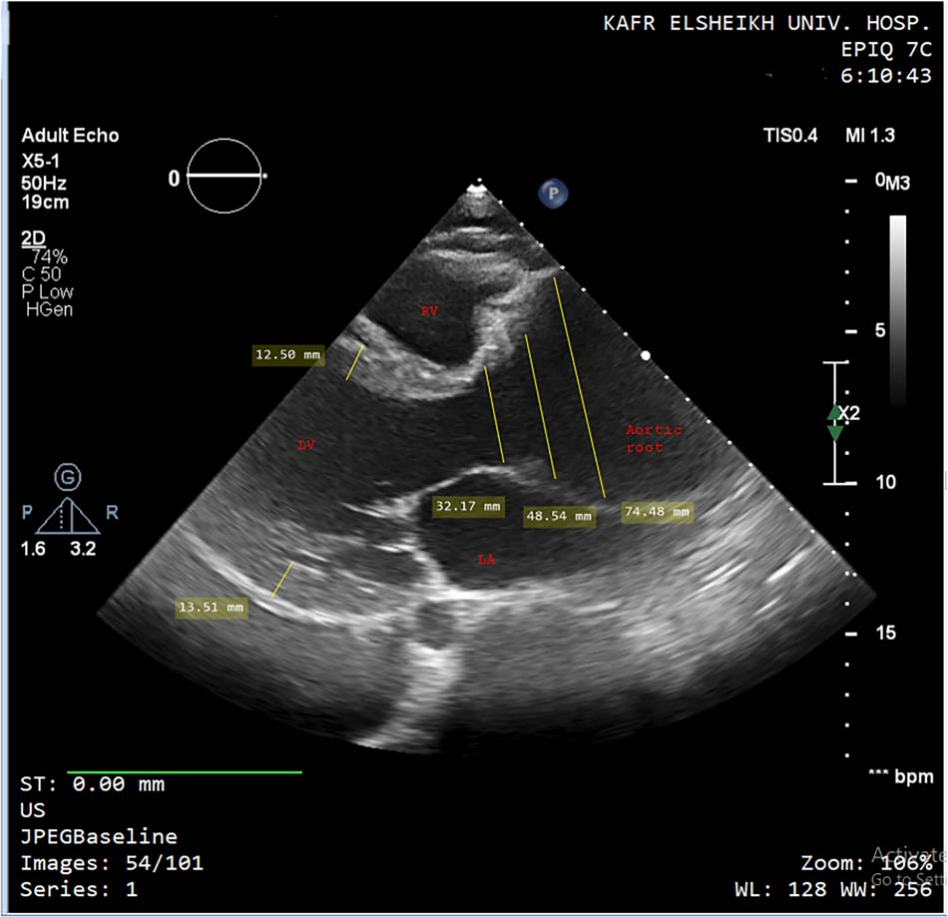
Transthoracic echocardiography showing the LV hypertrophy and the aortic root dilatation.

Laboratory findings were notable for an elevated level of Brain Natriuretic Peptide (BNP) (340 pg/mL). Additional findings included bilateral mild pleural effusion and cardiomegaly observed in the chest X-ray (Fig. 1). The ECG revealed sinus tachycardia with inverted T waves in leads V3 and V4 (Fig. 2). Transthoracic echocardiography (TTE) showed a mildly dilated and hypertrophied left ventricle (LV), slightly impaired LV systolic function with an ejection fraction of 48% by M-mode, impaired diastolic function, moderate mitral valve regurgitation, and severe aortic valve regurgitation. The aortic root was markedly dilated, with diameters of 3.2 cm at the annulus, 4.9 cm at the sinus of Valsalva, and 7.5 cm at the Sino tubular junction (STJ) (Fig. 3).

**Figure 4:**
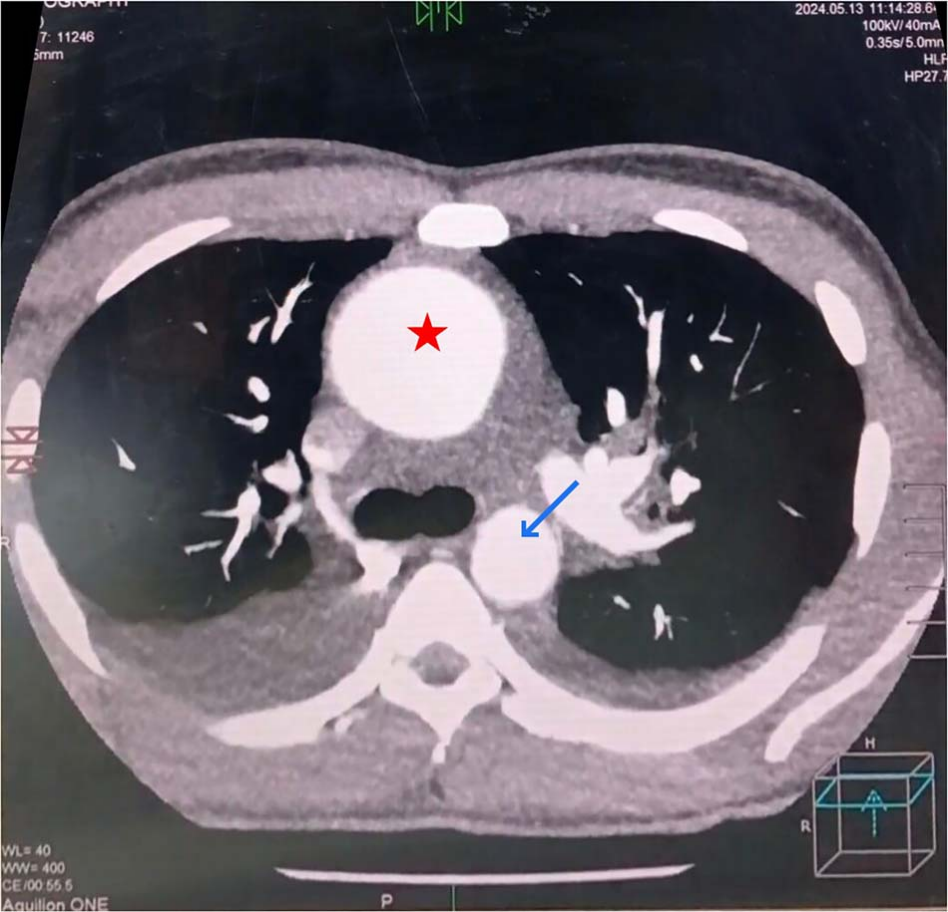
Transverse section of CT aortography showing the massively dilated ascending aorta (star) and descending aorta (arrow).

Consequently, CT aortography was performed, confirming the echocardiographic measurements of the aortic root diameters. Moreover, it revealed dilation of the ascending aorta with a diameter of 10 cm and the descending aorta with a diameter of 2.5 cm (Fig. 4). The patient was initiated on the following medications: Furosemide (20 mg every 8 hours), Ramipril (1.25 mg once daily), and Empagliflozin (10 mg once daily). Within 10 days, the patient experienced slight improvement in symptoms, particularly dyspnea, and reported better sleep. A follow-up chest X-ray showed partial resolution of the pleural effusion.

Initially, we considered connective tissue disease like Marfan or Ehlers-Danlos syndrome. However, the patient lacked the typical physical features and associated signs of these conditions. Additionally, his pulmonary function test was normal, which would likely be abnormal in a multisystem connective tissue disorder. Laboratory results showed normal ESR and C-reactive protein levels, reducing the likelihood of vasculitis as a cause of the aortic root dilatation. The autoimmune panel, including ANA and RF tests, was also negative.

Acromegaly was then considered as the potential cause of LV hypertrophy and aortic root dilation, especially considering his family history of the condition. Therefore, insulin-like growth factor 1 (IGF-1) level was ordered and was elevated (320 ng/ml), and the GH level remained above normal (1.1 ng/ml) after an oral glucose tolerance test confirming the diagnosis of acromegaly. A T2-weighted brain MRI image showed a 7 × 4 mm hyperintense mass in the Sella turcica, consistent with a pituitary microadenoma.

Subsequently, the surgery decision was taken after obtaining consent from the patient. Following a normal coronary angiography, the patient underwent a composite graft replacement procedure, involving the aortic valve, aortic root, and ascending aorta (Bentall Procedure). A mechanical valve was utilized, and the patient began taking Warfarin at a daily dose of 3 mg, with close monitoring to achieve a target INR of 2 to 3.

Following consultation with the endocrinologist, the patient began receiving monthly injections of Octreotide acetate. A comprehensive follow-up plan was established, which included monitoring the functioning of the valve, assessing LV function, ensuring medication compliance, and checking IGF-1 and INR values. Apart from mild exertional dyspnea, he remained asymptomatic for 8 weeks after the surgery. During this period, follow-up echocardiograms showed the replaced aortic valve functioning normally without any leakage or dysfunction. The aortic root and ascending aorta were also stable, with no signs of dilation, aneurysm, or other complications. There was no progression of his LV hypertrophy either. His most recent IGF-1 level was 290 ng/ml which is mildly reduced than the first, and he has been maintained on Octreotide without significant adverse effects.

## Discussion

This case report on a middle-aged patient with congestive heart failure symptoms and a massively dilated aortic root secondary to acromegaly emphasizes the importance of considering endocrine disorders in the differential diagnosis of cardiovascular structural abnormalities. The familial occurrence of acromegaly reflects the need for careful family history assessment, especially in rare presentations, and introduces a genetic dimension of acromegaly that warrants further exploration. One limitation of this report is its narrow scope and potential overlooking of other possible complications the patient might have experienced that needed longer follow up.

Acromegaly cases are usually sporadic. Very rarely, acromegaly may be inherited in either as part of multiple endocrine neoplasia type 1 or as a condition known as familial isolated pituitary adenoma. Recent studies have identified a few families where acromegaly is passed down through generations because of this condition and ongoing research is being conducted to better analyze its genetic basis [2].

Cardiac involvement in acromegaly is primarily characterized by concentric myocardial hypertrophy, affecting the interventricular septum and the posterior wall of the left ventricle. This can occur without hypertension and even in young patients under the age of 30. Congestive heart failure (CHF) may develop if cardiac conditions worsen, particularly if GH hypersecretion continues and other risk factors are present. At this stage, echocardiography may reveal varying degrees of cavity dilation [3]. In some cases of acromegaly, high-output heart failure may present with a slight reduction in ejection fraction at the time of diagnosis. The study by Damjanovic et al reported the incidence of overt heart failure at the time of diagnosis of acromegaly to be 10% [4].

Aortic root dilatation is another potential consequence of persistent elevation of IGF-1 and GH. The study by Casini et al conducted an echocardiography on 42 patients with acromegaly, comparing them to 42 matched controls based on age, sex, body surface area, and hypertension. The results showed that the average aortic root diameter at the level of the aortic leaflets was larger in the acromegaly patients (3.4 ± 0.5 cm) compared to the control group (2.9 ± 0.4 cm). Additionally, aortic ectasia, defined as an aortic root diameter ≥ 3.8 cm, was observed in 26.1% of acromegalic patients, whereas it was present in only 2.3% of the control subjects. However, in our case report, the aortic root diameter was significantly larger than the average measurements reported for acromegalic patients in the study by Casini et al [5]. To our knowledge, there is no other documented case with this degree of aortic root dilatation (7.5 cm at the STJ) at such a young age.

Similarly, in Van der Klaauw et al., aortic root diameters were assessed in 37 acromegalic patients (18 with active disease and 19 with controlled disease) using echocardiography over an observation period of 1.9 years. The results showed that the diameters of the aortic root at the STJ and ascending aorta were significantly larger in acromegaly patients compared to controls. Additionally, during follow-up, the diameters increased at the annulus and STJ. When analyzing patients with active and inactive disease separately, it was found that only the diameter of the STJ increased in patients with inactive acromegaly during follow-up. This study highlights the progressive nature of aortic root dilation in acromegaly patients, particularly at specific anatomical sites [6].

In the literature, there are a few case reports of acromegaly that were not initially identified through typical clinical symptoms but were instead discovered incidentally in asymptomatic individuals. For instance, the case report by Yeboah-Kordien et al describes a 60-year-old patient who was not previously known to have acromegaly, and his initial point-of-care ultrasound raised concerns about possible cardiac enlargement, leading to a formal echocardiogram that revealed significant aortic root dilation measuring 4.5 cm. Subsequent blood tests confirmed elevated IGF-1 levels. A brain MRI identified a focal lesion in the pituitary gland, which was surgically removed, confirming the diagnosis of a GH-secreting pituitary adenoma [7].

Massive aortic root dilation can lead to heart failure. When the aortic root dilates significantly, it can cause severe aortic insufficiency which results in LV volume overload and eventually heart failure. In the Cardiovascular Health Study, researchers explored the link between aortic root size and heart failure (HF). They found that individuals with larger aortic root dimensions had a higher risk of developing HF [8].

Surgery is often necessary for massive aortic root dilation with severe aortic regurgitation to prevent complications and improve heart function. The primary surgical options include Aortic Root Replacement (Also known as the Bentall procedure), where both the aortic root and the aortic valve are replaced with a composite graft. After undergoing the Bentall procedure, several long-term factors need to be considered. Patients will require lifelong anticoagulant therapy (in case of using a metallic valve) and regular follow-up appointments to monitor heart function and the condition of the graft and valve. It is crucial for patients to be informed about potential complications, such as graft infection, valve issues, or aortic dissection, and to seek immediate medical attention if any symptoms occur [9].

Normalization of GH secretion can improve structural and functional cardiac abnormalities in acromegaly patients. However, the long-term cardiac effects of treatment in those with CHF are not well researched. A retrospective review of 330 acromegaly patients in Bihan et al identified 8 cases with CHF diagnosed before, during, or after acromegaly in nine patients. Despite treatment, three patients had poor GH control, with two dying within five years. In contrast, five patients with good GH control showed stable or improved clinical status and quality of life. The study concluded that while short-term cardiovascular improvements were noted after treatment of acromegaly, the impact on long-term survival is uncertain [10].

Acromegaly caused by a pituitary microadenoma is typically treated through a combination of surgery, medication, and sometimes radiation therapy. The primary treatment is usually transsphenoidal surgery. However, the choice of treatment depends on numerous factors, including the size and location of the tumor [11]. In our case, the neurosurgeons decided against performing surgery because it was very small and close to the cavernous sinus. Therefore, Somatostatin analogue (Octreotide) was prescribed and a plan for regular follow up and close monitoring was made.

In conclusion, this case report emphasizes the value of considering genetic and familial factors in cardiac diagnoses and highlights the necessity for comprehensive diagnostic work-ups, including endocrine evaluations. Furthermore, the association between aortic root dilation and genetic or endocrinal conditions should be considered, particularly in such uncommon first-time presentations.
